# RhoA/ROCK-2 Pathway Inhibition and Tight Junction Protein Upregulation by Catalpol Suppresses Lipopolysaccaride-Induced Disruption of Blood-Brain Barrier Permeability

**DOI:** 10.3390/molecules23092371

**Published:** 2018-09-17

**Authors:** Shan Feng, Li Zou, Hongjin Wang, Ran He, Ke Liu, Huifeng Zhu

**Affiliations:** 1 College of Pharmaceutical Sciences and Chinese Medicine, Southwest University, 2# Tiansheng Road, Beibei District, Chongqing 400715, China; fengshan@swu.edu.cn (S.F.); zxm18611@163.com (L.Z.); wanghongjin0105@outlook.com (H.W.); hr606@swu.edu.cn (R.H.); liuke1030@126.com (K.L.); 2 Sichuan Vocational College of Health and Rehabilitation, Zigong 643000, China

**Keywords:** catalpol, LPS, BBB, tight junction, RhoA/ROCK2

## Abstract

Lipopolysaccaride (LPS) directly or indirectly injures brain microvascular endothelial cells (BMECs) and damages the intercellular tight junction that gives rise to altered blood-brain barrier (BBB) permeability. Catalpol plays a protective role in LPS-induced injury, but whether catalpol protects against LPS-caused damage of BBB permeability and the underlying mechanism remain to be delineated. Prophylactic protection with catalpol (5 mg/kg, i.v.) consecutively for three days reversed the LPS-induced damage of BBB by decreased Evans Blue (EB) leakage and restored tight junctions in C57 mice. Besides, catalpol co-administrated with LPS increased BMECs survival, decreased their endothelin-1, TNF-Α and IL-6 secretion, improved transmembrane electrical resistance in a time-dependent manner, and in addition increased the fluorescein sodium permeability coefficient of BMECs. Also, transmission electron microscopy showed catalpol protective effects on tight junctions. Fluorescence staining displayed that catalpol reversed the rearrangement of the cytoskeleton protein F-actin and upregulated the tight junction protein of claudin-5 and ZO-1, which have been further demonstrated by the mRNA and protein expression levels of ZO-1, ZO-2, ZO-3, claudin-5, and occludin. Moreover, catalpol concurrently downregulated the mRNA and protein levels of RhoA, and ROCK2, the critical proteins in the RhoA/ROCK2 signaling pathway. This study thus indicated that catalpol, via inhibition of the RhoA/ROCK2 signaling pathway, reverses the disaggregation of cytoskeleton actin in BMECs and prevents down-regulation of junctional proteins, such as claudin-5, occludin, and ZO-1, and decreases endothelin-1 and inflammatory cytokine secretion, eventually alleviating the increase in LPS-induced BBB permeability.

## 1. Introduction

**Figure 1:**
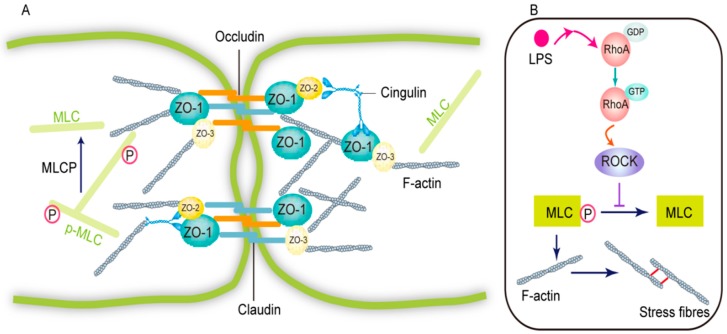
Tight junctions at the brain microvascular endothelial cells [1,2] and the mechanism associated with LPS increases the paracellular permeability [10,11,12]. (**A**) Tight junctions comprise transmembrane proteins (claudins and occludin), cytoplasmic attachment proteins (ZO-1, ZO-2, ZO-3), and cytoskeleton protein F-actin. (**B**) LPS stimulation of RhoA results in activation of ROCK. ROCK phosphorylates and inactivates myosin light chain phosphatase (MLCP), leading to myosin light chain (MLC) phosphorylation and activation, which further results in actin polymerization and F-actin stress fibers formation. Finally, cell contraction induces increased paracellular permeability.

The blood-brain barrier (BBB) is a major internal barrier in the human body. It comprises brain microvascular endothelial cells (BMECs), pericytes, astrocytic end-feet, basal laminar cells, neurons, and microglia. Among them, the tight junction between BMECs is the fundamental structure of the BBB, the first barrier to maintain homeostasis of the cerebral microenvironment [1]. The tight junction is composed of transmembrane proteins, cytoplasmic attachment proteins, and the cytoskeleton protein F-actin (Figure 1). The transmembrane proteins encompass three types of intact membrane proteins—occludin, claudin, and junction adhesion molecules (JAMs) [2]. The cytoplasmic attachment proteins encompass zonula occludens (ZO)-1, -2, and -3 proteins. The expression and spatial organization of these proteins are closely relevant to the BBB functions [3]. 

Lipopolysaccharide (LPS) is a type of glycoprotein localized on the outer membrane of Gram-negative bacilli, frequently used to induce a neuroinflammation model in rodents [4,5,6]. During the process, LPS stimulates a robust in vivo inflammatory response, directly or indirectly damaging BMECs, thereby altering BBB permeability and promoting pathogenesis and progression of cerebrovascular inflammation [7,8]. Tight junction destruction, endothelial damage, glycocalyx degradation, glia limitans breakdown, and astrocyte changes are the main mechanisms for LPS-induced disruption of BBB [3,9]. Rho-kinase (ROCK) belongs to the serine/threonine kinases family and is an important downstream effector of the small GTP-binding protein RhoA. The RhoA/ROCK signaling pathway is important in the regulation of permeability of vascular endothelial permeability. Despite the fact that pathogenic mechanism underlying LPS-induced elevation of vascular endothelial permeability remains to be clarified, some studies have revealed that the LPS-induced disruption of BMECs tight junction results from the activation of RhoA/ROCK signaling pathway [10,11,12]. LPS directly or indirectly (via cytokine) stimulation of Rho results in RhoA binding GTP (activation) while not binding GDP (inactivation), and then the ROCK is activated. ROCK phosphorylates and inactivates myosin light chain phosphatase (MLCP), leading to myosin light chain (MLC) phosphorylation and activation, which further results in activation of actinomyosin contractility and increased paracellular permeability. There are two isoforms of ROCK: ROCK1 and ROCK2. Owing to the fact ROCK2 is highly expressed relative to ROCK1 in all human and mouse brain cell types and particularly enriched in rodent brain endothelial cells and astrocytes compared to neurons, it is therefore the key isoform driving BBB impairment and brain endothelial damage [13].

Catalpol, the major active ingredient in *Rehmannia glutinosa*, plays a role in anti-inflammation [14,15,16], reducing capillary permeability [17], and reducing blood sugar [18,19]. Moreover, in vivo and in vitro studies have reported that catalpol exerts a protective role in LPS-induced trauma. In vitro, catalpol can protect dopaminergic neurons against LPS-induced neurotoxicity in mesencephalic neuron-glia cultures [20], antagonize apoptosis induced by LPS in PC12 cells [21], and alleviate inflammatory damage caused by LPS in astrocytes [15]. In vivo, catalpol may possess therapeutic potential against LPS induced acute systemic inflammation in mice [22], protect against LPS-induced acute mice lung [23] or rat liver injury [24], as well as ameliorate LPS-induced microvascular barrier damage and hemorrhage in rat [25]. Previous studies from our group demonstrated that catalpol markedly attenuated primary BMEC cell swelling, and prolonged BMEC survival [26]. Nonetheless, whether LPS-induced damage to BBB permeability could be prevented by catalpol and the underlying mechanism is yet to be elucidated. The present study found that catalpol exerted a protective role in the damage of LPS-induced BBB permeability by down-regulating the RhoA/ROCK2 signaling pathway as well as up-regulating the BMEC tight junction proteins. 

## 2. Materials and Methods

### 2.1. Chemicals and Antibodies

DMEM-F12 medium was from GIBCO (Invitrogen Corporation, Carlsbad, CA, USA). Catalpol was purchased from the National Institute for the Control of Pharmaceutical and Biological Products (Beijing, China). LPS (from *Escherichia coli*) (L-2630), was purchased from Sigma-Aldrich (St. Louis, MO, USA). Trizol reagent was obtained from Invitrogen. Rhodamine-conjugated phalloidin was purchased from Invitrogen (Molecular Probes, Eugene, OR, USA, R415). All the antibodies used in this study were from Cell Signaling Technology (Danvers, MA, USA) except the anti-β-actin and anti-ZO-1 antibodies which were from ZSGB-BIO (Beijing, China). The enzyme-linked immunosorbent assay (ELISA) kit of endothelin-1was purchased from ZSGB-BIO. PrimeScript RT reagent Kit with gDNA Eraser was purchased from Takara (Beijing, China). Power UP SYBR Green Master Mix was obtained from Thermo Fisher (Waltham, MA, USA).

### 2.2. Isolation and Purification of Rat BMECs

Primary rat BMECs were prepared from the brain of 2–3 weeks old SD rats as described in a previous report [27]. Briefly, meninges, large vessels, and white matter were removed carefully and the grey matter was minced into small pieces of approximately 1.0 mm^3^ in ice-cold D-Hanks buffer. After centrifugation at 150× *g* for 3 min, the precipitatate layer was added with trypsin (2.5 mg/mL) and digested at 37 °C for 1.5 h. Cold DMEM-F12 (1:1) medium with 10% FBS was added to terminate the digestion and then it was centrifuged at 150× *g* for 5 min. The precipitate was resuspended in 25% BSA and centrifuged at 600× *g* for 15 min. The micro-vessels obtained were then digested in collagenase type II (1.0 mg/mL) and DNase (1.5 mg/mL) at 37 °C for 1 h and the medium with 10% FBS was added to suspend it. This new suspension was filtered through a 10-mm-pore-size nylon mesh and washed with medium containing 10% FBS. After the filtrate was centrifuged at 150× *g* for 5 min, the precipitate layer was re-suspended with a medium with 20% FBS, basic fibroblast growth factor (bFGF, 1.0 ng/mL), heparin (100.0 mg/mL), penicillin (100.0 U/mL), L-glutamine (0.6 mg/mL) and streptomycin (100.0 mg/mL). After the density adjustment, the micro-vessel endothelial cells were seeded on 25 cm^2^ plastic dishes pre-coated with gelatin (15.0 mg/mL) and incubated at 37 °C and 5% CO_2_. The culture medium was changed every 2 days. When the confluence reached 80% (the 7th day), the endothelial cells were further purified with trypsin (2.5 mg/mL) and EDTA (0.2 mg/mL) solution. The purified endothelial cells were used in further experiments.

### 2.3. In Vivo LPS Challenge and Catalpol Protective Effect

**Figure 2:**
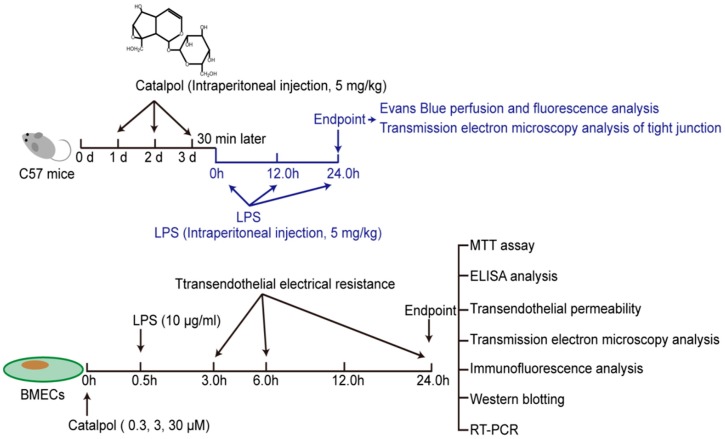
The study design of catalpol suppresses LPS-induced disruption of BMECs tight junction in vivo (**top**) and in vitro (**bottom**). BMECs, brain microvascular endothelial cells.

C57 male mice (10–14 weeks old) were purchased from the Experimental Animal Center, Chongqing Medical University (Chongqing, China) and housed in the Experimental Animal Center, College of Pharmaceutical Sciences & College of Chinese Medicine, Southwest University (Chongqing, China). All the experiments were performed in accordance with China’s Guidelines for Care and Use of Laboratory Animals. Mice were randomly grouped as animals undergoing a vehicle injection (Control); animals undergoing a LPS injection (LPS); animals undergoing a LPS injection and pre-treated with dexamethasone (LPS + Dex); animals undergoing a LPS injection and pre-treated with catapol (LPS + Cat). The dosage regimen was shown in Figure 2 (top). It is need to note that as dexamethasone is a common remedy for sepsis therapy, the present applied it as a positive therapy control.

Mice in LPS + Cat group were given intraperitoneal injection of 5 mg/kg catalpol, once a day for three days. After that, mice in all groups except Control were weighed and given a series intraperitoneal injections of 5 mg/kg LPS dissolved in sterile normal saline at 0, 12, 24 h. Dex group mice were given 40 μg/kg dexamethasone 30 min after the first dose of LPS. 4 h after the last dose of LPS, 3 mice in each group were intracardially perfused with prewarmed 0.9% NaCl briefly to wash out blood cells, followed by 0.5% Evans Blue in cold 4% PFA. After perfusion, the brain was taken out, post-fixed in 4% PFA at 4 °C for 4 h, and then cryoprotected in 20 and 30% sucrose solutions in PBS at 4 °C for 3 days. The brain tissues were cut 30 μm in thickness in a cryostat (Leica Microsystems, Wetzlar, Germany). Sections were mounted on gel-coated slides, dried at 37 °C for 1 h, and kept at −20 °C for use. The Evans Blue-perfused cerebral microvessels in the sections, which exhibit fluorescence when the Evans Blue binds to proteins, were examined and imaged using a Leica fluorescence microscope equipped with a CCD camera (Leica Microsystems).

Besides, another 3 mice in each group were anesthetized and perfused transcardially with 2.5% glutaraldehyde in 0.1 M PBS. Approximately 1 mm^3^ of the brain tissue was taken and fixed in freshly prepared 3% glutaraldehyde for 4 h at 4 °C and post-fixed in 1% osmium tetroxide for 2 h. The specimens were then dehydrated through a graded series of ethanol solutions (50%→70%→80%→90%→95%→100%), and embedded in Epon 812 overnight. 60–80 nm ultrathin brain sections were obtained and stained with uranyl acetate and lead citrate. Sections were then examined using transmission electron microscopy (Hitachi-H7500, Tokyo, Japan). And the length of Chinese fir ridge structure was analyzed by Image J.

### 2.4. In Vitro LPS Challenge and Catalpol Protective Effect

During the preliminary experiment, the dose and treatment time of LPS were studied. The results indicated that 10 μg/mL and 24 h were suitable dose and treatment time for LPS challenge studies. BMECs were pre-treated with or without catalpol (fasudil). After 30 min, LPS was added to wells and were treated for 24 h (catalpol final concentration: 0.3, 3, 30 μM, fasudil final concentration: 25 μM, LPS final concentration: 10 μg/mL). All the reagent were dissolved in PBS. What’s more, as the purpose of the in vitro experiments is to elucidate underlying mechanism, the present study applied fasudil as positive control.

#### 2.4.1. MTT Assay for Cell Viability

BMECs viability was determined with MTT assay. Each group was assigned with 6 wells and cells at a density of 1 × 10^5^ cells per well. Cells were seeded in 96 well plate (coated with 0.1% polylysine) for 100 μL/well, after 12 h, catalpol (0.3, 3, 30 μM) and fasudil (25 μM) were added for pre-incubation. 30 min later, cells were further treated with LPS for 24 h. The cells were incubated with 5 mg/mL MTT during the last 24 h of LPS challenge. After that, the medium was removed and formazan salts dissolved with 150 μL of dimethylsulfoxide. The absorbance values were determined at 570 nm with an automatic multi-well spectrophotometer (Bio-Rad Laboratories, Hercules, CA, USA, 168–9520). The experiment was repeated 3 times in each group.

#### 2.4.2. Measurement of Transendothelial Electrical Resistance (TEER)

The TEER values of BECMs at different LPS challenge time were measured by an epithelial-volt-ohm resistance meter (ERS-2, Millipore, Burlington, MA, USA) according to the protocol provided by the company. Briefly, BMECs were seeded at 2.0 × 10^5^ cells/cm^2^ on the apical side of a 1.1 cm^2^ polyethylene terephthalate filter insert with 4.0 μm porosity (Corning 3460), coated with 0.1% polylysine. For culture chambers (12-well plates), 1.5 mL completed culture media were added and cultured for 2 days (to reach confluence) prior to addition of test compounds. In addition, according to previous report [28], the minimum TEER values were set at 70 Ω × cm^2^ before applying the barrier for further experiments. Each group was assigned with 6 wells. The treatment plan was described in Section 2.4. After the treatment, the background TEER value was measured. The final result was calculated as the TEER value of different cells subtracted of the corresponding background TEER value and then multiplied by the area of insert membrane. The values are shown as Ω × cm^2^.

#### 2.4.3. Determination of the Transendothelial Permeability by Sodium Fluorescein

The flux of sodium fluorescein across the endothelial cells was determined after the measurement of TEER value. Briefly, the culture medium in the inserts was replaced with 0.5 mL DMEM-F12 medium containing 100 μg/mL sodium fluorescein. After 2 h incubation, the samples were taken from the well, and the absorbance of the samples was measured by fluorospectrophotometer.

#### 2.4.4. Immunofluorescence Analysis

BMECs (400 μL) were seeded at 5.0 × 10^5^ cells/mL on 35 mm glass bottom dish coated with 0.1% polylysine, and cultured for 2 days to reach confluence. The treatment plan was described in Section 2.4. After treatment, cells were first washed with PBS and fixed with 4% paraformaldehyde for 30 min at room temperature. After the removal of excessive paraformaldehyde, the fixed cells were incubated in a fresh blocking buffer (0.5% Triton X-100 in PBS, pH 7.4, containing 10% normal goat serum) for 1 h at room temperature. The cells were incubated overnight at 4 °C with anti-claudin-5 (Thermo Fisher Catalog # 34-1600, Waltham, MA, USA) or anti-ZO-1 (Proteintech Catalog (Rosemont, IL, USA): 20742-1-AP) primary antibody, which were diluted 1:50 (anti-claudin-5) or 1:20 (ZO-1) in PBS with 3% bovine serum albumin. The primary antibodies were detected by incubation with Alexa Fluor 488 (1:300) in PBS containing 3% bovine serum albumin. Cells were then incubated with phalloidin-594 (Invitrogen) for 30 min at room temperature in the dark. After that, the cell nucleus was stained with 4, 6-diamidino-2-phenylindole (DAPI). Images were captured on an Olympus FV1200 laser scanning confocal microscopy (Olympus, Tokyo, Japan).

#### 2.4.5. ELISA Analysis

BMECs (200 μL) were seeded at 1.0 × 10^5^ cells/mL on 96 well plates coated with 0.1% polylysine, and cultured for 2 days to reach confluence. The treatment plan was described in Section 2.4. The contents of endothelin-1, TNF-Α, and IL-6 of the supernatant fraction was detected by sandwich ELISA assay according to the manufacturer’s instructions, respectively. Absorbance was measured at 495 nm using a microplate ELISA reader (BioTek, Winooski, VT, USA). Each final value was quantified against a standard curve calibrated with known amounts of protein.

#### 2.4.6. Transmission Electron Microscopy Analysis of Tight Junction

BMECs (500 μL) were seeded at 5.0 × 10^5^ cells/mL on 35 mm dish coated with 0.1% polylysine, and cultured for 2 days to reach confluence. After treatment described in Section 2.4, BMECs were prefixed with 2.5% glutaraldehyde in 0.1 M PBS at 4 °C overnight, post-fixed in 1% buffered osmium tetroxide, dehydrated in graded alcohols, embedded in Epon 812, sectioned with ultramicrotome and stained with uranyl acetate and lead citrate. The intercellular tight junction and autophagy precursors of the cells were observed with a transmission electron microscopy (Hitachi-H7500).

#### 2.4.7. RT-PCR and qPCR

Total RNA was extracted from BMECs using the Trizol reagent as the manufacturer’s instructions. Pure RNA was then reverse-transcribed using SuperScript III Reverse Transcriptase (Invitrogen) along with gene-specific primers, and PCR was performed using PrimeScript RT reagent Kit. Quantitative PCR was performed using PowerUP SYBR Green Master Mix and the Bio-rad CFX-96 real-time PCR instrument (Bio-Rad). In all cases β-actin was used as an internal control.

#### 2.4.8. Western Blot Analysis

BMECs were scraped and collected in 1 mL cell lysis buffer (50 mM Tris-HCl PH 7.4, 2% SDS, 0.1 M NaCl, 1 mM EDTA, 1% triton X-100, 0.5 μg/mL aprotinin, 1 mM sodium rothovanadate, 1 mM PMSF). Then, each sample was incubated on ice for 20 min and centrifugated at 12,000× *g* for 15 min. The supernatant of each culture was freeze-dried using vacuum freeze-drying apparatus after added with the protease inhibitors. Then, 10–20 μg of total proteins were separated on a 10% Tris/Glycine SDS-PAGE gel and subsequently transferred to PVDF membrane. Following 45 min incubation in a PBST containing 5% non-fat milk, the blots were probed with specific antibodies including anti-Claudin-5, anti-ZO-1, anti-RhoA, anti-ROCK2, anti-β-actin. The bound primary antibodies were detected by horseradish peroxidase conjugated secondary antibodies accordingly. The activity of peroxidase on the blot was visualized by enhanced chemiluminescence (ECL) detection reagents (Millipore). The blots were quantified by Quantity One software (Bio-Rad). The concentration of the loaded cellular proteins was normalized against the internal control β-actin and then the value was expressed as each normalized data relative to control.

### 2.5. Statistics

Data was drawn from at least three separate experiments performed in triplicate. The data were presented as mean ± SD and analyzed using the SPSS version 19.0 statistical software (International Business Machines Corporation, New York, NY, USA). Comparisons between multiple groups were performed by one-way analysis of variance procedures coupled with Bonferroni’s post hoc tests. The histologic semi-quantitative analysis was compared by the nonparametric Mann-Whitney test. *p* value < 0.05 was considered significant.

## 3. Results

### 3.1. Catalpol Alleviated the Reduction in LPS-Induced BBB Permeability

Evans Blue (EB) binds to albumin, and hence, cannot traverse through normal BBB, whereas the structure-damaged BBB allowed EB to pass. 

**Figure 3:**
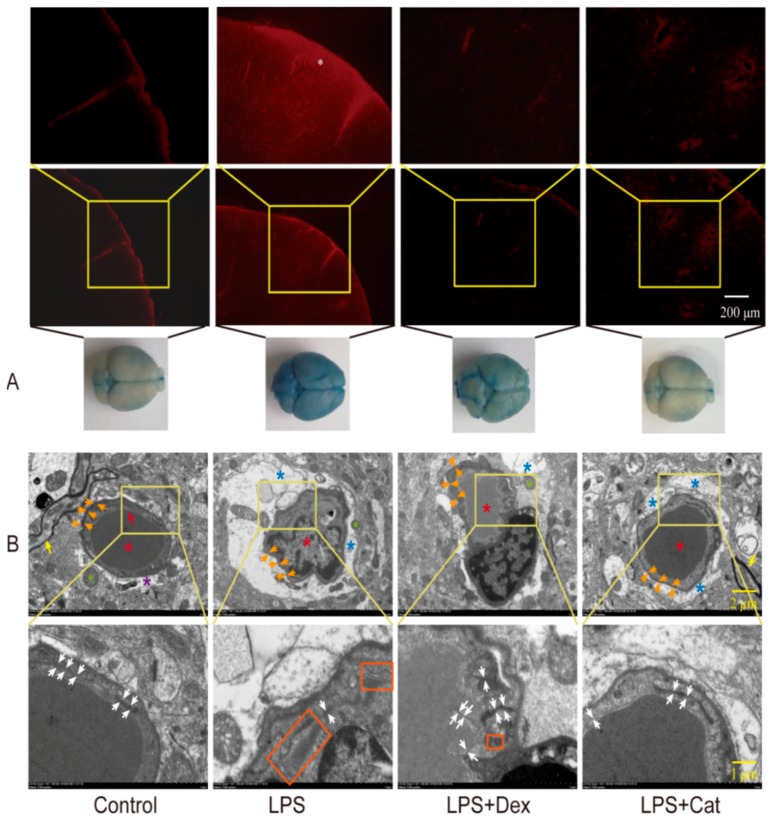
Catalpol improved LPS-induced BBB damage. (**A**) EB leakage in brain tissue. *, the cerebral cortex was filled with robust fluorescent signals. (**B**) the ultrastructural changes of the BBB. Red asterisks indicate the vessel; Red arrows indicate the endothelial cells; Orange arrows indicate the basal lamina; Purple asterisks indicate the astroglial end feet; Green asterisks indicate the axon or dendrite; Yellow arrows indicate the myelin sheath; Blue asterisks indicate the swollen astroglial end feet; White arrows indicate the tight junction; Red panels indicate the detached tight junction. LPS, mice given a series intraperitoneal injection of 5 mg/kg LPS dissolved in sterile normal saline at 0, 12, 24 h. LPS + Dex, mice were given 40 μg/kg dexamethasone 30 min after the first dose of LPS. LPS + Cat. Before administration of LPS, mice were given intraperitoneal injection of 5 mg/kg catalpol, once a day for three days.

Therefore, the damage status of BBB structure can be reflected by the fluorescence intensity of EB in cerebral slides. When 15 mg/kg of LPS was administered to C57 mice within 24 h, the mouse brain was conspicuously stained in blue, and the whole cerebral cortex was filled with robust fluorescent signals, with maximal fluorescence on the cortex surface (Figure 3A). Besides, the fluorescence intensity was significantly increased in comparison to control group (1433.33 ± 305.05 vs. 250 ± 88.88, *p* < 0.05). However, 5 mg/kg catalpol markedly attenuated the blue staining in the mouse brain, as reflected by the fluorescent intensity of EB decreased to 310 ± 87.17 (*p* < 0.05) (Figure 3A). Moreover, in the positive drug dexamethasone group, the blue staining in the mouse brain was not conspicuous, and the reduction in the EB leakage was significant compared to LPS (fluorescence intensity at 1050.00 ± 278.38). The ultrastructural changes of the BBB were also examined (Figure 3B). Images from control group identified the BBB unit composed of endothelial cells, basal lamina, pericytes (not very clear), and astrocyte end feet. After the LPS injury, the basement membrane was disrupted and tight junctions were damaged, as well as evident swollen astrocyte end feet. However, in the catalpol-treated and dexamethasone-treated groups, the basement membrane was preserved and the tight junctions were restored.

### 3.2. BMEC Characterization and Screening for LPS Concentration and Treatment Time

**Figure 4:**
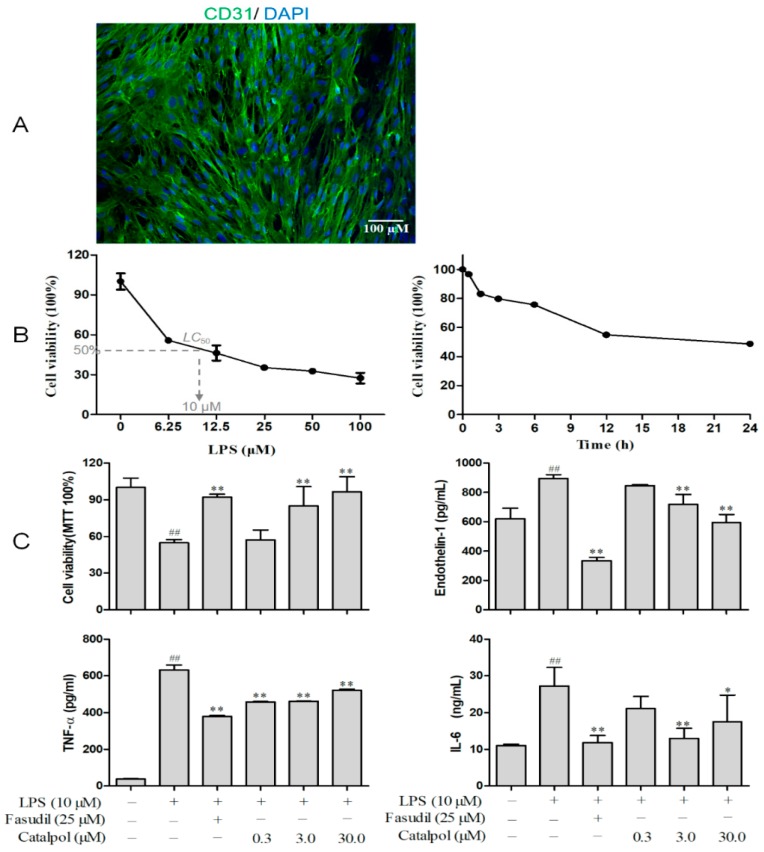
Catalpol improved BMEC survival and its secretory function on endothelin-1 and inflammatory cytokines. (**A**) BMECs characterization via immunocytochemical staining with FITC labeled CD31. (**B**) LPS concentration and treatment time screening. (**C**) The effects of catalpol on cell survival rate (MTT test), endothelin-1, TNF-α and IL-6 secretion of LPS injured BMECs. (±SD, *n* = 6), ^##^ *p* < 0.01 vs. the control group; * *p* < 0.05, vs. the model group; ** *p* < 0.01, vs the model group. BMECs, brain microvascular endothelial cells.

CD31 is one of the specific markers for vascular endothelial cells, and it can be utilized to identify cell origin and purity. As shown in Figure 4A, BMEC cells, purified in the current study, were labeled with fluorescent-tagged CD31, green color, and 98% purity was found, rendering them eligible for subsequent experiments (Figure 4A). After 24 h of stimulation with LPS, the growth of BMECs was inhibited in a concentration-dependent manner, with an approximate LC_50_ value of 10 µg/mL (Figure 4B). Therefore, 10 µg/mL LPS was selected for establishing the BMEC damage model (this concentration significantly inhibited the BMEC cell survival rate, while not severely damaging the cells, thus allowing an adequate number of cells to be collected for subsequent experiments). The optimal intervention time required for LPS to establish a BMEC damage model was estimated according to the determined LPS concentration. As shown in Figure 1B, LPS inhibited the cell growth in a time-dependent manner, and only 50% of the cells survived the 24-h time point. Therefore, in this study, the BMEC damage model was established utilizing 10 µg/mL LPS for 24 h.

### 3.3. Catalpol Improved BMEC Survival and Its Secretory Function

MTT tests demonstrated that 3 and 30 μM catalpol dramatically reversed the LPS-induced damage and improved the cell survival (*p* < 0.01), while catalpol at 0.30 μM concentration did not exhibit any significant effect (Figure 4C). In addition, after LPS intervention for 24 h in BMECs, endothelin-1 secretion was elevated from 620.44 ± 39.28 to 895.44 ± 36.93 pg/ML (*p* < 0.01), whereas it was declined to 846.00 ± 11.00, 717.67 ± 96.64, and 594.33 ± 77.78 pg/mL by 0.3, 3, and 30 μM catalpol, respectively, in a concentration-dependent manner (Figure 4C). Similarly, the production of inflammatory cytokines TNF-Α and IL-6 were significantly increased (20-fold for TNF-α and 2.7-fold for IL-6) 24 h after treatment with LPS (Figure 4C). This is strongly suggestive of an LPS-dependent inflammatory injury. Besides, the increase of inflammatory cytokines could be reversed by catalpol (0.3–30 μM) while without concentration-dependent manner. Moreover, ROCK inhibitor, fasudil at 25.0 μM robustly improved the cell survival and decreased endothelin-1, TNF-Α and IL-6 secretion (Figure 4C).

### 3.4. Catalpol Alleviated the Reduction in LPS-Induced BMEC Permeability

**Figure 5:**
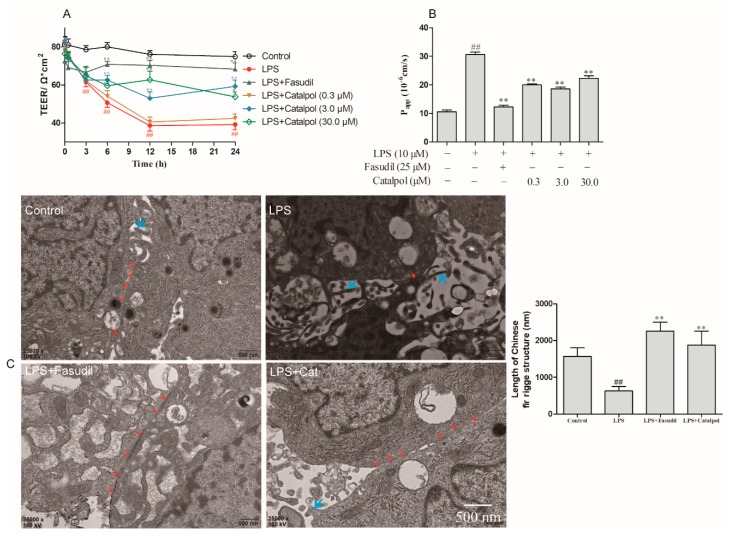
The protective effects of catalpol on TEER, fluorescein sodium transmittance, and tight junction of BMEC. (**A**) Catalpol (3.0 and 30.0 μM) increases LPS-induced reduces of TEER (Ω·cm^2^). (**B**) Catalpol (0.3, 3.0 and 30.0 μM) reduces LPS-induced increase of the transmittance of fluorescein sodium (Papp, 10^−6^ cm/s). (**C**) Catalpol (3.0 μM) improves LPS-induced injury of tight junction as detected by transmission electron-microscopy. Red arrows, the tight junction (Chinese fir ridge structure) between BMECs; Blue arrows, plasma membrane protrusions. ^##^ *p* < 0.01 vs. the control group; ** *p* < 0.01, vs. the model group. (±SD, *n* = 6).

After BMEC cells were cultured with LPS, the transmembrane electrical resistance was reduced in a time-dependent manner, beginning after 3 h of incubation (Figure 5A). The catalpol intervention at 3.0 and 30.0 μM remarkably reversed the reduction in transmembrane electrical resistance and enhanced the intercellular tightness of BMECs at the time points of 6, 12, and 24 h (Figure 5A). Moreover, fluorescein sodium penetration test confirmed that catalpol (0.3–30 μM) reversed the increase in the permeability coefficient after 24 h BMECs’ culture with LPS (Figure 5B). However, the dose-dependent manner has not been observed in fluorescein sodium penetration test, which indicated that other ways such as endocytosis may mediate it across the BMECs.

### 3.5. Catalpol Antagonized the Changes in the Ultrastructure of BMEC Tight Junction Caused by LPS Trauma

Under the physiological condition of BMECs, the tight junction was observed by electron microscopy, and continuous strip-like high electron-dense Chinese fir ridge structure (the complexity and integrity of the tight junction were reflected by the number and length of Chinese fir ridge structures) was detected (Figure 5C). In addition, plasma membrane protrusions, namely villous structures, were detected in most cells. Upon LPS trauma, the tight junction of BMEC was not continuous, with the shorter version of Chinese fir ridge structure (Figure 5C). Catalpol (3.0 μM) and fasudil (25 μM) alleviated the LPS-induced damage of the BMEC tight junction (Figure 5C). 

### 3.6. Catalpol Antagonized F-Actin Disaggregation and Downregulation of the LPS-Induced Expression of ZO-1 and Claudin-5

**Figure 6:**
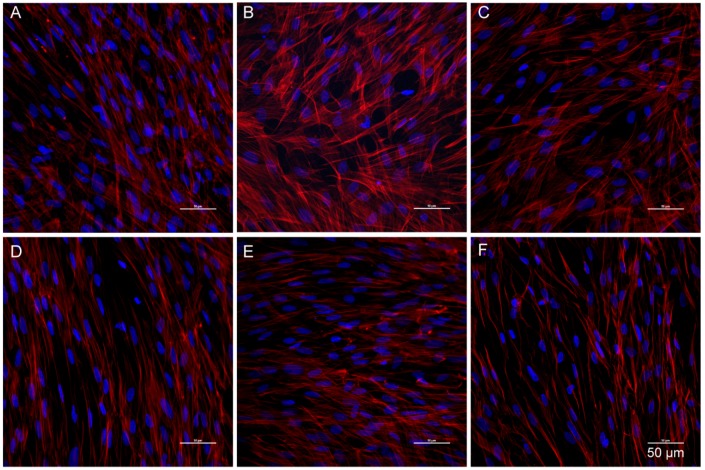
The effects of catalpol on F-actin polymerization induced by LPS detected by immunofluorescence staining. (**A**) normal group; (**B**) model group; (**C**) Fasudil group; (**D**) catalpol 0.3 μM group; (**E**) catalpol 3.0 μM group; (**F**) catalpol 30.0 μM group.

**Figure 7:**
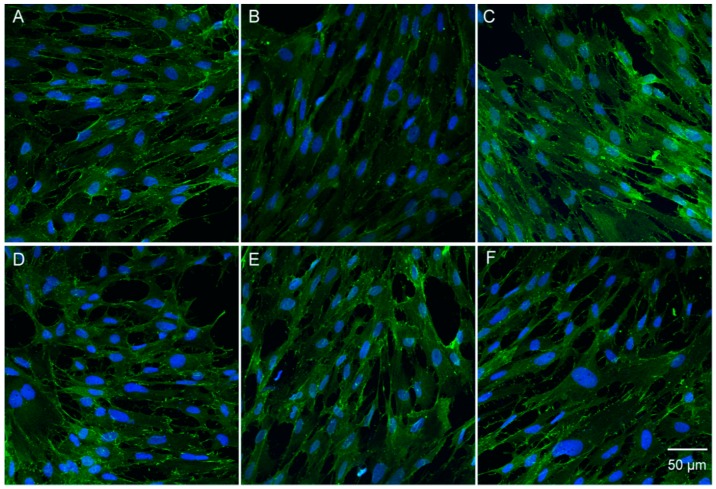
The effects of catalpol on ZO-1 decreased expression induced by LPS detected by immunofluorescence staining. (**A**) normal group; (**B**) model group; (**C**) Fasudil group; (**D**) catalpol 0.3 μM group; (**E**) catalpol 3.0 μM group; (**F**) catalpol 30.0 μM group.

**Figure 8:**
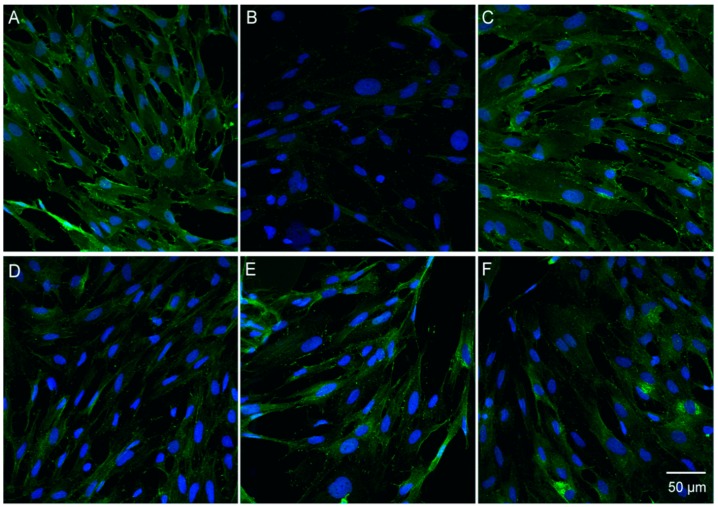
The effects of catalpol on Claudin-5 decreased expression induced by LPS detected by immunofluorescence staining. (**A**) normal group; (**B**) model group; (**C**) Fasudil group; (**D**) catalpol 0.3 μM group; (**E**) catalpol 3.0 μM group; (**F**) catalpol 30.0 μM group.

Physiologically, F-actin was distributed on the cellular edge, forming a few actin ribbons, without any visible stress fibers. Also, the protein line was intact and continuous, without any obvious cracks, but with a distinct boundary (Figure 6A); ZO-1 and claudin-5 were distributed on the surface of the cell membrane in a continuous pattern (Figure 7A and Figure 8A). After incubation of BMECs with LPS for 24 h, the nuclear pyknosis, as well as the cell outline, were blurred and prone to disintegration and dissipation (Figure 7B and Figure 8B). In addition, the red fluorescence of F-actin was enhanced with obvious cracks and visible stress fibers, as well as serrated fractures were presented in the actin ribbon (Figure 6B). Besides, the green fluorescence-labeled that ZO-1 was detected only in a few cells (Figure 7B); whereas, any claudin-5 expression was not detected (Figure 8B). Upon prophylactic protection with catalpol (0.3, 3.0, and 30.0 μM) incubation for 24 h, the BMEC cell number increased remarkably. Moreover, the red fluorescence of F-actin decreased, and cytoskeleton was restored, in addition to the upregulation of the expression of claudin-5 and ZO-1 on the cell membrane and the appearance of intercellular junction (Figure 6, Figure 7 and Figure 8).

### 3.7. Catalpol Antagonized Changes in the Protein Expressions Relevant to BMEC Tight Junction and RhoA/ROCK Signaling Pathway Caused by LPS Trauma

**Figure 9:**
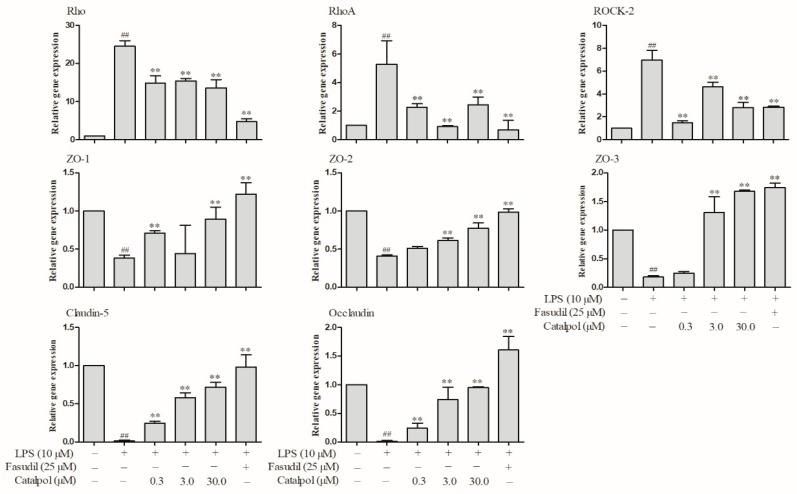
The effects of catalpol on the changes of tight junction and Rho/ROCK pathway mRNA level induced by LPS detected by qPCR (±SD, *n* = 3). ^##^ *p* < 0.01 vs. the control group; ** *p* < 0.01, vs. the LPS group.

After incubation of BMECs with LPS, a significant reduction in the mRNA levels was detected in the tight junction proteins: ZO-1, ZO-2, ZO-3, claudin-5 and occludin (*p* < 0.01, Figure 9). 

**Figure 10:**
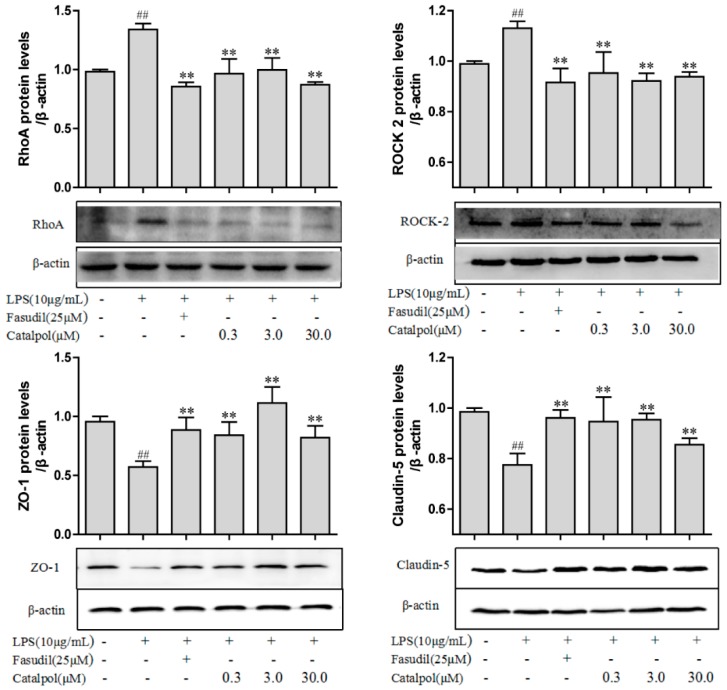
The effects of catalpol on the changes of tight junction and Rho/ROCK pathway proteins expression induced by LPS detected by qPCR (±SD, *n* = 3). ^##^ *p* < 0.01 vs. the control group; ** *p* < 0.01, vs. the LPS group.

Moreover, only a significant increase in the mRNA levels in Rho, RhoA, and ROCK2 was observed (*p* < 0.01, Figure 9). The prophylactic protection with catalpol or fasudil led to a marked reversion of the above changes in the mRNA levels (*p* < 0.01, Figure 9). Western blotting (WB) detection also demonstrated a significant reduction in the protein levels of ZO-1 and claudin-5 after LPS trauma in BMECs and a significant increase in the protein levels of RhoA and ROCK-2 (*p* < 0.01, Figure 10). The prophylactic protection with catalpol or fasudil led to a marked reversion of the above changes in the protein levels (*p* < 0.01, Figure 10). 

## 4. Discussion

The aim of this study was to explore whether LPS-induced damage to BBB permeability could be prevented by catalpol and the underlying mechanism. Results showed that catalpol exerted a protective role in the damage of LPS-induced BBB in C57 mice, which may be explained by its inhibition of the RhoA/ROCK2 signaling pathway, reversing the disaggregation of cytoskeleton actin and preventing down-regulation of junctional proteins, such as claudin-5, occludin, and ZO-1 in BMECs. 

Previous studies demonstrated that several inflammatory mediators and cytokines activate RhoA/ROCK signaling pathway, and phosphorylate the myosin light chain to elevate myosin activity, thereby increasing the actin-myosin cross-linkage, as well as, F-actin contraction and aggregation, causing cell trauma [3,29,30,31]. Essler et al. discovered that LPS caused HUVEC contraction via the activation of Rho/ROCK signaling pathway [32]. Liu et al. revealed that LPS activated the Rho/ROCK signaling pathway, thereby causing PMVECs trauma and cytoskeleton rearrangement [33]. Fang et al. also reported that Rho/ROCK signaling pathway activation was primarily responsible for BMEC trauma and LPS-increased permeability [11]. In this study, catalpol downregulated the mRNA and proteins levels of the critical molecules—Rho, RhoA, and ROCK2—in RhoA/ROCK2 signaling pathway, indicating that the protective activity of catalpol in BMECs tight junction is correlated with the inhibition of RhoA/ROCK2 signaling pathway. What’s more, it is reported that endothelin-1 and inflammatory cytokines are also involved in the BBB integrity destruction. For example, intrastriatal injection of endothelin-1 could increase blood-brain barrier permeability both in rats and dogs while the mechanism is not clear [34,35,36]. TNF-α, IFN-γ, IL-1, and IL-6 could induce permeability increase via down-regulation of tight junction proteins or actin restructuring in endothelial cells [37,38,39]. However, McKenzie further pointed out that the gradual increase in permeability induced by TNF-α does not mediate by Rho, ROCK, and MLCK, but involves long-term reorganization of tight junction proteins [37]. In the present study, the increase of endothelin-1 and inflammatory cytokines TNF-α and IL-6 could be reversed by catalpol (0.3–30 μM). Taken together, the decrease of endothelin-1 and inflammatory cytokines secretion could be another mechanism for catalpol ameliorating LPS-induced tight junction destruction.

LPS is a common proinflammatory element. In the systemic inflammatory response, it increases the endothelial cell permeability and microvascular permeability, reduces the effective circulating blood volume, aggravates hock, and eventually leads to organ function damage [1,5,6,8,9]. Presently, effective clinical measures to harness the high vascular permeability are lacking. Therefore, based on the antibiotics and hormones, identifying an adjuvant drug for decreasing the vascular permeability is under intensive research for the prevention and treatment of systemic inflammation. Catalpol is an iridoid compound with a broad spectrum of pharmacological activities, involving multiple signaling pathways. It activates PI3K/Akt/Bad signaling pathway and blocks the MAPK signaling pathway to exert the effect of anti-apoptosis in neurons and endothelial cells [21,40]. In addition, it modulates angiogenesis via the activation of a JAK2/STAT3 signaling pathway and upregulates synapsin expression via the activation of BDNF/Trk B signaling pathway for the benefit of the degenerative nerve diseases [26,41]. Furthermore, catalpol inhibits the transcriptional activity of NF-κB, thereby declining the expression of proinflammatory molecules: MCP-1, TNF-α, iNOS, and AGE receptor of RAGE [15]. The current study further demonstrated that catalpol, via blocking the RhoA/ROCK2 signaling pathway, inhibits cytoskeleton actin disaggregation in BMECs and upregulates the expressions of the tight junction proteins, claudin-5, occludin, and ZO-1, which eventually alleviates the LPS-increased BBB permeability. Therefore, in the LPS-induced systemic inflammation, catalpol, via targeting multiple pathways, is expected to be a potential valuable therapy for patients with sepsis.

Pharmacokinetic characters have great influence on the effects of drugs, especially for those targeted on brain diseases. Pharmacokinetic study reflected that catalpol (i.v., 6 mg/kg) rapidly transported into the cerebrospinal fluid (CSF) and exhibited a peak concentration of 676 ng/mL (equal to 1.87 μM) at 5 min post-administration in rats, with AUC_CSF_/AUCplasma at 5.8% [42]. According to the above pharmacokinetic characters, it is clear to see that the in vivo dose (5.0 mg/kg) was closely related to the in vitro concentration of 3.0 μM in the present study, and then the protective effects of catalpol in vivo and in vitro are corelated. Besides, the same report showed that catalpol was eliminated rapidly, with a half-life (t1/2) at 1.5 h in cerebrospinal fluid, and 0.7 h in plasma [42]. Due to its rapid in vivo elimination (t1/2 = 1.5 h), it is seemed that catapol administrated two days before exposure to LPS make no sense in the present study. In another word, only the last dose of catapol may have exhibited protective effects on the LPS-induced injury. Hence, the animal administration scheme of catalpol should be further evaluated according to its pharmacokinetic characters (especially in the sepsis model) and effective concentration. 

Fasudil was approved for the treatment of cerebral vasospasm in Japan and China. In the present study, fasudil, a positive ROCK inhibitor, displayed better protective effects than catapol. In fact, previous reports showed that fasudil could prevent LPS-induced heart oxidative stress, abnormal F-actin distribution, and oxidative phosphorylation, which concur to improve cardiac contractile and bioenergetic function in guinea pigs [43]. Meanwhile, the same protective effects have also been observed in septic liver injury [44]. It is then suggested that fasudil may be also a valuable therapy for sepsis. According to the above discussion, searching for an adjuvant drug is a clinical measure for sepsis therapy based on the antibiotics and hormones. However, whether co-administrated of the ROCK inhibitors (such as fasudil, catalpol) and hormones (dexamethasone) has synergistic effects on sepsis therapy is unknown, and further studies are needed to conduct to validate this hypothesis.

## 5. Conclusions

This study indicated that catalpol, by inhibiting the RhoA/ROCK2 signaling pathway, reverses the disaggregation of cytoskeleton actin in BMECs and prevents down-regulation of junctional proteins, such as claudin-5, occludin, and ZO-1, as well as decreases endothelin-1 and inflammatory cytokines secretion, eventually alleviating the increase in LPS-induced BBB permeability.
